# Safety of artemisinins in first trimester of prospectively followed pregnancies: an observational study

**DOI:** 10.1016/S1473-3099(15)00547-2

**Published:** 2016-05

**Authors:** Kerryn A Moore, Julie A Simpson, Moo Kho Paw, MuPawJay Pimanpanarak, Jacher Wiladphaingern, Marcus J Rijken, Podjanee Jittamala, Nicholas J White, Freya J I Fowkes, François Nosten, Rose McGready

**Affiliations:** a Centre for Epidemiology and Biostatistics, Melbourne School of Population and Global Health, The University of Melbourne, Melbourne, VIC, Australia; b Macfarlane Burnet Institute for Medical Research and Public Health, Melbourne, VIC, Australia; c Shoklo Malaria Research Unit, Mahidol-Oxford Tropical Medicine Research Unit, Faculty of Tropical Medicine, Mahidol University, Mae Sot, Thailand; d Mahidol-Oxford Tropical Medicine Research Unit, Faculty of Tropical Medicine, Mahidol University, Bangkok, Thailand; e Centre for Tropical Medicine and Global Health, Nuffield Department of Medicine, University of Oxford, Oxford, UK; f Department of Epidemiology and Preventive Medicine and Department of Infectious Diseases, Monash University, Melbourne, VIC, Australia

## Abstract

**Background:**

Artemisinins, the most effective antimalarials available, are not recommended for falciparum malaria during the first trimester of pregnancy because of safety concerns. Therefore, quinine is used despite its poor effectiveness. Assessing artemisinin safety requires weighing the risks of malaria and its treatment. We aimed to assess the effect of first-trimester malaria and artemisinin treatment on miscarriage and major congenital malformations.

**Methods:**

In this observational study, we assessed data from antenatal clinics on the Thai–Myanmar border between Jan 1, 1994, and Dec 31, 2013. We included women who presented to antenatal clinics during their first trimester with a viable fetus. Women were screened for malaria, and data on malaria, antimalarial treatment, and birth outcomes were collected. The relationship between artemisinin treatments (artesunate, dihydroartemisinin, or artemether) and miscarriage or malformation was assessed using Cox regression with left-truncation and time-varying exposures.

**Findings:**

Of 55 636 pregnancies registered between 1994 and 2013, 25 485 pregnancies were analysed for first-trimester malaria and miscarriage, in which 2558 (10%) had first-trimester malaria. The hazard of miscarriage increased 1·61-fold after an initial first-trimester falciparum episode (95% CI 1·32–1·97; p<0·0001), 3·24-fold following falciparum recurrence (2·24–4·68; p<0·0001), and 2·44-fold (1·01–5·88; p=0·0473) following recurrent symptomatic vivax malaria. No difference was noted in miscarriage in first-line falciparum treatments with artemisinin (n=183) versus quinine (n=842; HR 0·78 [95% CI 0·45–1·34]; p=0·3645) or in risk of major congenital malformations (two [2%] of 109 [95% CI 0·22–6·47] versus eight (1%) of 641 [0·54–2·44], respectively).

**Interpretation:**

First-trimester falciparum and vivax malaria both increase the risk of miscarriage. We noted no evidence of an increased risk of miscarriage or of major congenital malformations associated with first-line treatment with an artemisinin derivative compared with quinine. In view of the low efficacy of quinine and wide availability of highly effective artemisinin-based combination therapies, it is time to reconsider first-trimester antimalarial treatment recommendations.

**Funding:**

The Wellcome Trust and The Bill & Melinda Gates Foundation.

## Introduction

The associations between first-trimester malaria, treatment, and miscarriage remain poorly documented because these events often occur before women present to antenatal care.^1^, ^2^ A single first-trimester malaria episode is associated with miscarriage,^3^ and women with first-trimester malaria who are not adequately treated are at high risk of placental malaria.^4^ Because women are usually not protected by preventive interventions until the second trimester,^3^ early diagnosis and effective treatment of first-trimester malaria are essential to limit the deleterious effects of malaria.^5^

Artemisinin derivatives (hereafter referred to as artemisinins) are the most effective antimalarials available. Artemisinin-based combination therapies are recommended by the WHO for first-line treatment of falciparum malaria, except during the first trimester of pregnancy.^6^ Animal studies have raised concerns about the safety of artemisinins in the first trimester, but data for human beings are scarce. In animals, artemisinins are embryotoxic and teratogenic because they deplete embryonic erythroblasts, causing miscarriage and congenital malformations (mainly cardiovascular and skeletal).^7^ If artemisinins are also embryotoxic or teratogenic in human beings, the embryo-sensitive period is predicted to be between 6 weeks' and 13 weeks' gestation when erythroblasts are the primary form of circulating red blood cells.^8^ Because of these safety concerns, quinine is still recommended for uncomplicated first-trimester falciparum malaria rather than artemisinins, despite being an inferior treatment.^6^ Available data for first-trimester artemisinin safety comes from observational studies of inadvertent treatments, which are common but rarely documented.^2^ No specific adverse effects have been noted in human beings in 935 documented first-trimester artemisinin treatments (appendix p 1),^9^, ^10^, ^11^, ^12^, ^13^, ^14^, ^15^, ^16^, ^17^, ^18^ which although reassuring, has not been sufficient to change treatment recommendations.^6^

The Shoklo Malaria Research Unit (SMRU) screens pregnant women frequently for malaria because there are no effective preventive interventions (appendix p 3).^19^ Since 1986, prospective data have been collected on confirmed malaria infections, antimalarial treatment, and pregnancy outcomes of women attending SMRU antenatal clinics, providing an important source of observational evidence on first-trimester artemisinin safety. In this setting, a single first-trimester malaria episode (falciparum or vivax) increased the odds of miscarriage, but first-trimester artemisinin treatment was not associated with miscarriage.^3^ However, for analytic clarity in this earlier study women with recurrent infections were excluded, which reduced the number of artemisinin treatments to 44 and overestimated the effect of malaria, because a recurrent infection in pregnancy depends on the fetus surviving the initial infection. Here, we extend this seminal study by including women with recurrent malaria, which might be either novel, recrudescent, or a relapse in the case of vivax malaria, and added 3 further years of data. Assessment of the safety of artemisinins requires weighing the risks of malaria and its treatment. Therefore, we sought to assess the effect of both first-trimester malaria and artemisinin treatment on miscarriage and major congenital malformations.

**Research in context**

**Evidence before this study**

We searched Scopus and PubMed for articles published up to Oct 5, 2015, in any language, that addressed the association between first-trimester artemisinin treatment and miscarriage using the search terms: “malaria or plasmodium”, “pregnan*”, “*artemisinin* OR ACT* OR artesunate OR artemether OR Coartem”, “first-trimester OR ‘first trimester’ OR ‘early pregnancy’”, and “miscarriage* OR abortion”. One study reported on the association between a single first-trimester malaria episode and miscarriage. No randomised controlled trials of first-trimester artemisinin treatment were identified. No studies have reported on the association between recurrent first-trimester malaria and miscarriage. Ten observational studies of first-trimester artemisinin treatment were identified totalling 935 documented treatments, and a systematic review published in 2007. These studies showed no evidence of an increased risk of miscarriage or major congenital malformations associated with first-trimester artemisinin treatment. Importantly, only one published study examining the association between first-line artemisinin treatment and miscarriage accounted for left truncation, which is necessary when women present at varying gestations due to the declining risk of miscarriage as a pregnancy progresses, and few were able to account for confounding by indication and disease severity.

**Added value of this study**

Assessment of the safety of artemisinin derivatives requires weighing the risks of falciparum malaria against those of its treatment. We noted that first-trimester falciparum malaria increases the risk of miscarriage, especially after recurrence. However, there was no evidence that first-line treatment with an artemisinin derivative in the first trimester was associated with an increased risk of miscarriage or congenital malformations compared with first-line quinine, which is currently recommended by the WHO. We compared first-line treatment with an artemisinin derivative with first-line quinine in women with first-trimester falciparum malaria in an area of low seasonal transmission, and accounted for confounding by indication and disease severity, thereby separating the effects of infection from the effects of treatment. To the best of our knowledge, this study is the first to estimate the association between recurrent first-trimester malaria and miscarriage, and contributes a further 183 documented first-trimester artemisinin treatments. Left truncation, which adjusts for the temporally changing risks of miscarriage and varying gestation at presentation, was also accounted for and is essential to avoid significant bias.

**Implications of all the available evidence**

Effective treatment of first-trimester falciparum malaria is imperative. Our results add to a growing body of observational evidence that artemisinins, the most effective antimalarials available, are safe in the first trimester of pregnancy.

## Methods

### Study design and participants

In this observational study, we assessed data from antenatal clinics on the Thai–Myanmar border between Jan 1, 1994, and Dec 31, 2013. We included women who presented to antenatal clinics during their first trimester with a viable fetus. Women were screened for malaria, and data for malaria, antimalarial treatment, and birth outcomes were collected. The Oxford Tropical Research Ethics Committee granted ethical approval for audits of SMRU clinical records (OXTREC 28-09), and the Tak Province Community Ethics Advisory Board provided local permission (T-CAB-4/1/2015). Data for first-trimester malaria from some of the records included in this analysis have been published previously.^3^, ^19^, ^20^, ^21^, ^22^

### Procedures

At SMRU antenatal clinics, women are encouraged to present early and return weekly throughout their pregnancy for malaria screening, consisting of a finger-prick blood sample that is examined by trained microscopists using Giemsa stained thick and thin blood films (appendix p 3).^5^ Women are also encouraged to present if they feel unwell, and to deliver at SMRU clinics. The first consultation involves taking obstetric and medical histories, a detailed clinical examination, and gestational age estimation.^23^ With each positive screen, information about species, parasitaemia, symptoms, and treatment are recorded. Women are also asked about recent antimalarial treatments at outpatient clinics, and these treatments (usually mefloquine–artesunate [MAS] for *P falciparum*) are recorded retrospectively. Presumptive malaria treatment is not used, and pregnancy termination is unavailable.

First-trimester non-malaria febrile morbidity was defined as fever (temperature ≥37·5^o^C) not associated with malaria. Malaria was defined as the presence of asexual stages of plasmodia parasites in the peripheral blood, counted per 500 white blood cells or 1000 red blood cells. Hyperparasitaemia was defined as 4% parasitaemia or greater, and severe malaria was defined according to signs of vital organ dysfunction. Symptomatic malaria was defined as patent parasitaemia and a history of fever (past 48 h) or temperature 37·5^o^C or greater. Vivax malaria was treated with oral chloroquine. Falciparum malaria was treated with oral quinine in the first trimester, or an artemisinin-based treatment in the second and third trimester (either artesunate, artemether–lumefantrine, dihydroartemisinin–piperaquine, or mefloquine–artesunate). Mefloquine monotherapy was given for falciparum malaria until 1996. Clindamycin was added to quinine and artesunate 7-day treatments in 2007 to augment efficacy. According to WHO recommendations, artemisinins were given in the first trimester for quinine failures, hyperparasitaemia, severe malaria, or if the fetus was no longer viable.^6^ Details on treatment regimens and drug manufacturers are given in the appendix (p 4).

### Outcomes

Primary exposures were malaria and first-line artemisinin treatment in the first trimester, defined as less than 14 weeks' gestation. The primary outcome was miscarriage, defined as fetal death before 28 weeks' gestation because infant respiratory support is unavailable. The ability to determine gestation and fetal viability at SMRU improved after ultrasound was introduced in 2002 (appendix p 3).^23^, ^24^ The date of miscarriage was recorded consistently as the date of expulsion of the uterine contents, either spontaneously or through surgical intervention, which can occur some time after intrauterine death. The secondary outcome was major congenital malformations. A surface examination was done on all newborns by trained staff; a physician verified all malformations, except for some early neonatal deaths. Artemisinin-based treatments were first deployed in the general population in 1994. Therefore, we included women who presented to antenatal clinics during their first trimester with a viable fetus between Jan 1, 1994, and Dec 31, 2013.

### Statistical analysis

We used Cox proportional hazards models accounting for left truncation (appendix p 5) and time-varying exposures for all miscarriage analyses, with censoring at the gestation time of miscarriage, gestation time when last seen, or 28 weeks' gestation. To assess the association between malaria and miscarriage, women entered the analysis at the gestation time of their first antenatal visit. Multivariable models adjusted for year of first consultation, gravidity, smoking, and first-trimester non-malaria febrile morbidity. To assess the association between first-line artemisinin treatment and miscarriage (primary analysis), we included women with first-trimester falciparum malaria, and compared first-line quinine treatment (including quinine plus clindamycin) with first-line mefloquine monotherapy, artemisinin treatment (all derivatives) following quinine failure (ie, artemisinin rescue), and first-line artemisinin treatment (all derivatives). Women entered the analysis at the gestation time of their first falciparum malaria episode. Treatments given after determination of fetal non-viability were excluded. Multivariable models adjusted for year of first consultation, disease severity pertaining to the first falciparum malaria episode (asymptomatic, symptomatic, or hyperparasitaemic or severe), and first-trimester non-malaria febrile morbidity. The prevalence of major congenital malformation was described by first-trimester falciparum malaria and first-line treatment. Malformations were grouped by organ system to increase the likelihood of detecting teratogenic signals.^25^ Data were analysed with Stata version 13 (StataCorp, College Station, TX, USA).

### Role of the funding source

The funding sources (The Wellcome Trust and The Bill & Melinda Gates Foundation) had no role in the study design, data collection, data analysis, data interpretation, writing of the report, or the decision to submit for publication. The corresponding author had full access to all data and had the final responsibility in the decision to submit for publication.

## Results

**Figure 1:**
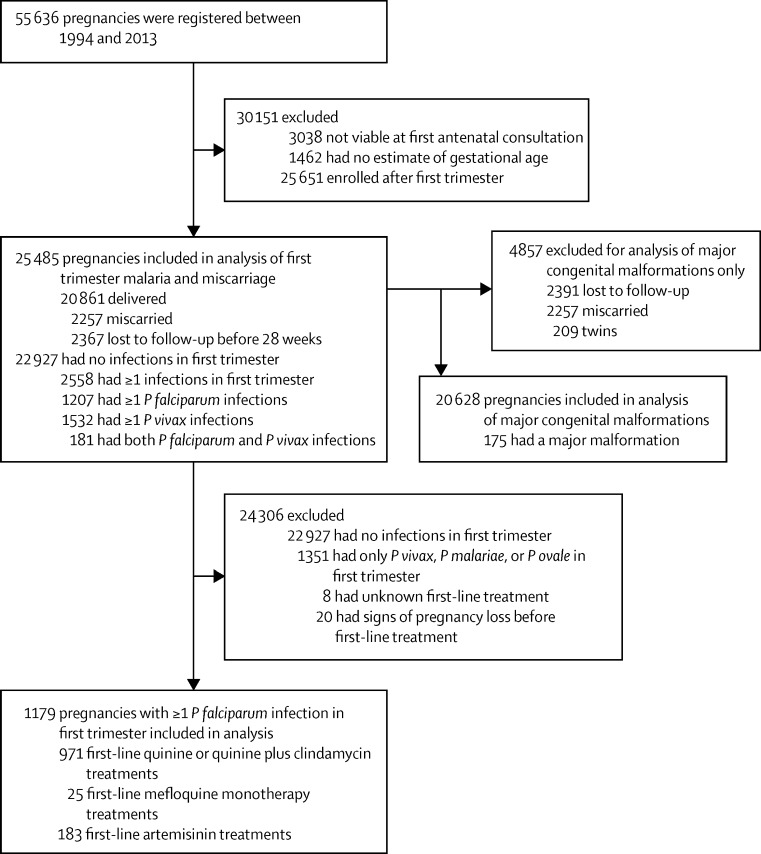
Study profile *P vivax*=*Plasmodium vivax. P malariae*=*Plasmodium malariae. P ovale*=*Plasmodium ovale. P falciparum=Plasmodium falciparum.*

Between Jan 1, 1994 and Dec 31, 2013, 55 636 pregnant women presented to SMRU clinics, of whom 25 485 (46%) presented during their first trimester with a viable fetus (figure 1). Of these, 2257 (10%) of 23 118 miscarried, 2367 (9%) of 25 485 were lost to follow-up before 28 weeks gestation, and 2558 (10%) of 25 485 had first-trimester malaria (figure 1). Women with first-trimester malaria were more likely to miscarry or be lost to follow-up and tended to present for antenatal care earlier, be younger, be primigravid, and smoke compared with women without first-trimester malaria (all p<0·0001; table 1).

**Figure 2:**
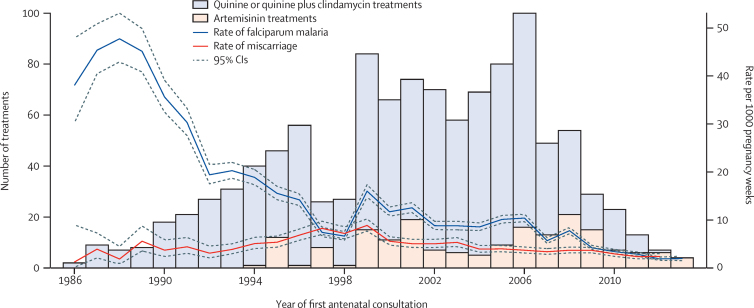
Frequency of first-line quinine and artemisinin treatments in first trimester and rates of falciparum malaria during pregnancy and miscarriage over time The increase in the rate of falciparum malaria in 1998 was due to the establishment of Shoklo Malaria Research Unit antenatal clinics in migrant communities.

Of the 2558 women with first-trimester malaria, 1207 (47%) had falciparum malaria, 1532 (60%) had vivax malaria, and 181 (7%) had both vivax and falciparum (either separate or mixed infections). Recurrent first-trimester falciparum malaria occurred in 162 (13%) of 1207 women, and recurrent first-trimester vivax malaria in 139 (9%) of 1532. Most (971 [80%] of 1207) women with first-trimester falciparum malaria were treated initially with quinine and 183 (15%) of 1207 were treated initially with artemisinin (ie, first-line artemisinin treatment; table 1). Of the 971 women who received first-line quinine treatment, 129 (13%) were rescued with artemisinin (usually artesunate monotherapy or artesunate plus clindamycin) following recurrence. Of the 183 first-line artemisinin treatments, 37 (20%) were for hyperparasitaemia (administered orally) or severe disease (administered parenterally). First-line treatment of first-trimester falciparum malaria occurred at a median of 8·2 gestation weeks (IQR 5·3–11·1). Loss to follow-up was similar between antimalarial treatment groups, except women receiving mefloquine–artesunate were less likely to be lost (p=0·0417; appendix p 6). Rates of falciparum malaria during pregnancy and miscarriage and the frequency of first-line quinine and artemisinin treatments in first trimester over time are shown in figure 2.

**Figure 3:**
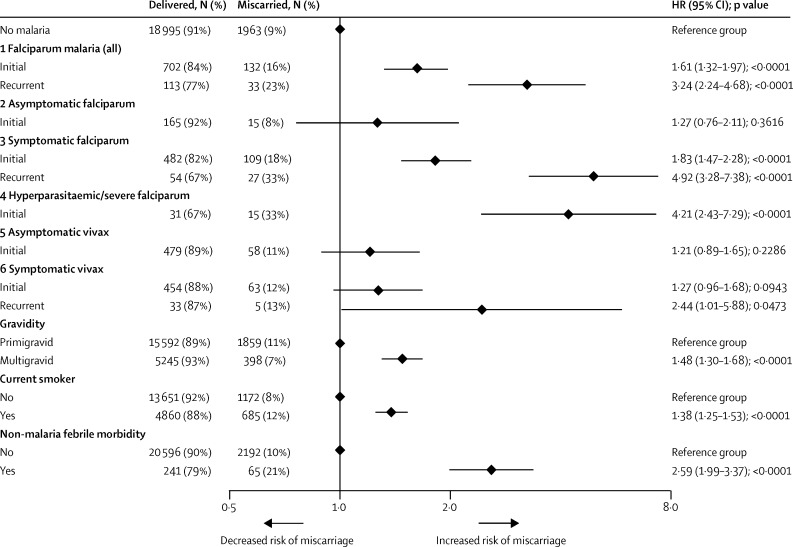
Association between initial and recurrent first-trimester malaria and miscarriage Models include women lost to follow-up before 28 weeks (until gestation time last seen), but percentage calculations for delivered or miscarried do not. Models for falciparum malaria (1–4) include women that might have also had first-trimester vivax, malariae, or ovale malaria. See appendix p 8 for associations in women with only first-trimester falciparum malaria. Models for vivax malaria (5–6) exclude women who also had first-trimester falciparum malaria. Models 2 and 5 exclude women with symptomatic malaria. Models 3 and 6 exclude women with asymptomatic infections. Model 4 excludes women with uncomplicated infections. Models were adjusted for year (by stratification due to non-proportional hazards [p<0·001]), gravidity, current smoking status, and non-malaria febrile morbidity in first trimester. Age and previous miscarriage were omitted from multivariable models due to collinearity with gravidity. Adjusted results for gravidity, current smoking status, and febrile morbidity in first trimester are shown from Model 1. 146 women had recurrent first-trimester falciparum malaria (136 had two and ten had three episodes). 13 women had recurrent (two) asymptomatic first-trimester falciparum malaria. 81 women had recurrent symptomatic first-trimester falciparum malaria (75 had two, and six had three episodes). 17 women had recurrent (two episodes) asymptomatic fi0rst-trimester vivax malaria, and none miscarried. 38 women had recurrent (two) symptomatic first-trimester vivax malaria. See appendix p 8 for a table version of this figure, including univariable associations.

Of 1207 women with first-trimester falciparum malaria, 165 (17% of 983 followed until 28 weeks') miscarried and 224 (19%) were lost to follow-up compared with 1963 (9%) of 20 978 and 1949 (9%) of 22 927 in women with no first-trimester malaria, respectively. In multivariable analyses, the hazard of miscarriage increased 1·61-fold (95% CI 1·32–1·97; p<0·0001) with an initial first-trimester falciparum malaria episode, and 3·24-fold (2·24–4·68; p<0·0001) with recurrent first-trimester falciparum malaria (figure 3). This association was stronger in women with symptomatic falciparum malaria than in women with asymptomatic falciparum malaria (figure 2). A single first-trimester hyperparasitaemic or severe falciparum malaria episode increased the hazard of miscarriage 4·21-fold (95% CI 2·43–7·29; p<0·0001; figure 3). An initial first-trimester vivax malaria episode, either asymptomatic or symptomatic, increased the hazard of miscarriage slightly (figure 3). Recurrent symptomatic first-trimester vivax malaria increased the hazard of miscarriage 2·44-fold (95% CI 1·01–5·88; p=0·0473; figure 3).

**Figure 4:**
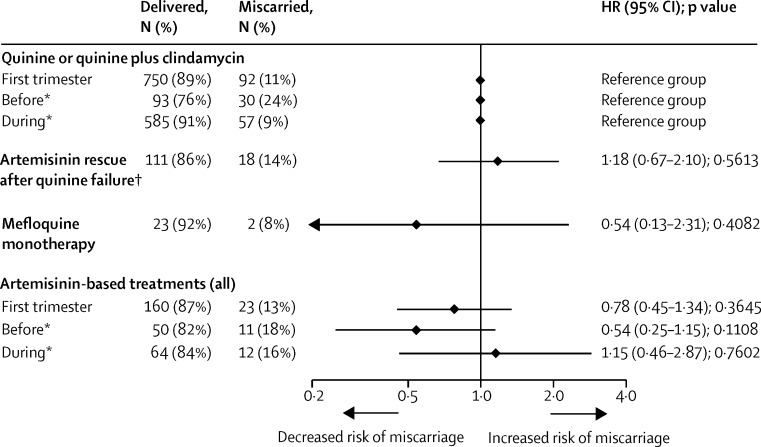
Association between first-line treatment of first-trimester falciparum malaria and miscarriage (n=1179) Models were adjusted for severity of the first falciparum malaria episode (asymptomatic, symptomatic, or hyperparasitaemic or severe), non-malaria febrile morbidity in the first trimester, and year of first consultation. See appendix p 9 for a table version of this figure, including univariable associations. *Hazard ratios are shown for treatments occurring before (<6 weeks’ gestation) and during (≥6 and <13 weeks’ gestation) the embryosensitive window. †Artemisinin rescue after quinine failure refers to artemisinin-based treatment in first trimester following failure of first-line treatment with quinine or quinine plus clindamycin. No miscarriages occurred in women who received artemisinin treatment after the embryo-sensitive window (≥13 and <14 weeks’ gestation). We did a subgroup analysis excluding women with asymptomatic malaria (n=919); the association between artemisinin treatment and miscarriage changed by <5% (appendix p 9). We did a subgroup analysis in women attending before 2007 whose gestational age was estimated from ultrasound biometry, because the accuracy of gestational age estimates affects the accuracy of the gestation time of antimalarial treatment, and quinine plus clindamycin succeeded quinine monotherapy in 2007 (n=469); associations were in the same direction but of greater magnitude (appendix p 9).

Of the 1207 women with first-trimester falciparum malaria, 1179 (98%) had a known first-line antimalarial treatment and a viable fetus at the time of treatment (figure 1). Most (842 [71%] of 1179) received first-line quinine (including quinine plus clindamycin), 129 (11%) of 1179 received first-line quinine followed by artemisinin (artemisinin rescue), 25 (2%) of 1179 received first-line mefloquine monotherapy, and 183 (16%) of 1179 received first-line artemisinin. First-line artemisinin treatment was not associated with miscarriage when compared with women who received first-line quinine only (HR 0·78 [95% CI 0·45–1·34]; p=0·3645; figure 4). Five (3%) of 183 women received two first-trimester artemisinin treatments; one miscarried, and four delivered.

Because animal studies suggest a theoretical embryo-sensitive window in human beings of 6–13 weeks' gestation, we also estimated the association between first-line artemisinin treatment and miscarriage before, during, and after this window.^9^ First-line artemisinin treatments before the embryo-sensitive window were associated with a non-significant decrease in the hazard of miscarriage (HR 0·54 [95% CI 0·25– 1·15]; p=0·1108), whereas treatments during the embryo-sensitive window were not associated with a changed hazard of miscarriage (1·15 [0·46–2·87]; p=0·7602; figure 4).

Of note, a high proportion of women who received first-line mefloquine–artesunate miscarried (15 [21%] of 71), and most miscarriages in the artemisinin treatment group (15 [63%] of 23) were in women who received mefloquine–artesunate specifically. Further, a third of women who received mefloquine–artesunate during the embryo-sensitive window miscarried (eight [33%] of 24). However, this might be explained by the circumstances of this treatment: women received mefloquine–artesunate from outpatient clinics (rather than antenatal clinics) where they presented because of illness before they became aware of their pregnancy. Additionally, mefloquine–artesunate treatments at outpatient clinics were given at earlier gestations than for the other treatments when women are at greater risk of miscarriage (mefloquine–artesunate: 3·8 weeks [IQR 1·9–7·4]; quinine: 10·1 [7·5–11·8]; other artemisinins: 12·4 [9·0– 13·3]; appendix p 7).

20 628 women presented for antenatal care between 1994 and 2013 and gave birth to a singleton newborn, of whom 175 (1%) of 20 628 (95% CI 0·73–0·98) had newborns with a major congenital malformation (figure 1). The prevalence of congenital malformations in the newborn babies of women with no first-trimester malaria was 0·84% (158/18 803 [95% CI 0·71–0·98]; table 2). The prevalence of congenital malformations was similar in the newborns of women with first-trimester vivax malaria (0·59% [95% CI 0·22–1·27]). In the newborn babies of women with uncomplicated first-trimester falciparum malaria, malformation prevalence was slightly higher (1·29% [10/773]; [95% CI 0·62–2·37]), but did not differ between the newborn of women who received first-line quinine (1·25% [8/641; [95% CI 0·54–2·44]) and those of women who received first-line artemisinin (2/109 [microphthalmia; imperforate anus]; 1·83% [95% CI 0·22–6·47]; p=0·7551; table 2). Two other newborns of mothers who had hyperparasitaemic or severe first-trimester falciparum malaria had a malformation (syndactyly; cleft lip and palate; 9·09% [2/22]; [95% CI 1·12–29·16]); both were of mothers who received first-line artemisinin, but only eight women received first-line quinine (table 2). No newborns of mothers who received artemisinin in first trimester had the skeletal or cardiovascular malformations as reported in animal studies.

## Discussion

First-trimester falciparum malaria increases the risk of miscarriage, especially after recurrence, but this large prospective observational study found no evidence that first-line treatment with an artemisinin derivative was associated with an increased risk of miscarriage or congenital malformations. Assessment of the safety of artemisinin treatment during pregnancy requires weighing the risks of falciparum malaria against those of its treatment. This is the first study to estimate the effects of initial and recurrent first-trimester malaria, its symptomatology, and its treatment on miscarriage.

Legitimate ethical concerns regarding randomised-controlled trials of first-trimester artemisinin treatment have meant that only observational studies have been done to date, and these have not adjusted for confounding by indication and disease severity in assessing risks and benefits.^26^ A major strength of this study is that it was possible to adjust for these important confounders by comparing with nearly 1000 women who received quinine treatment (appendix p 5). Left truncation, which adjusts for the temporally changing risks of miscarriage and varying gestation at presentation, was also accounted for since this is essential to avoid bias.^27^ Nevertheless, this study still has limitations common to observational designs. Data were collected over a long period of time, relatively few first-trimester artemisinin treatments were given, toxicities other than miscarriage and major malformations detectable at birth from surface examination were not captured, and all artemisinin derivatives were analysed together. Several associations of considerable magnitude had wide CIs that crossed null, and we cannot rule out potential confounding effects of time and unmeasured variables, or residual confounding by disease severity. Furthermore, women with first-trimester malaria were more likely to be lost to follow-up, raising the possibility of informative right censoring, but this would underestimate the effect of malaria (appendix p 5).

We noted no evidence that first-line treatment with an artemisinin derivative increased the rate of miscarriage compared with first-line treatment with quinine. There was a higher risk of miscarriage in women who received an artemisinin derivative during the putative embryo-sensitive window, but this might be explained at least in part by the administration of mefloquine–artesunate at earlier gestations to symptomatic women in the routine outpatient clinics compared with the active surveillance of antenatal clinics. In rats, embryotoxicity of artesunate was attenuated when co-administered with mefloquine.^28^ Primates, including human beings, might be less sensitive to the effects of artemisinins because of differences in placentation and the visceral yolk sac, which could result in different levels of embryonic exposure to artemisinins.^7^, ^29^ Additionally, a 3-day artemisinin regimen means that the exposure period is relatively short in human beings because organogenesis is 3 days in rats but 3 months in human beings.^7^, ^29^ Therefore, artemisinin-induced depletion of embryonic erythroblasts severe enough to cause miscarriage in rats might not translate to human beings, but could still cause congenital malformations.

We cannot draw firm conclusions on the possible effects of first-trimester artemisinin treatment on congenital malformations because of relatively small numbers of treatments and cases. Furthermore, the prevalence of major congenital malformations is most likely an underestimation because only those detectable at birth from surface examination and heart auscultation were recorded routinely, and major malformations (particularly cardiovascular) are often not detected or confirmed until later in life. Only four newborns whose mother received first-line artemisinin treatment during first trimester had a major congenital malformation, and the organ systems involved were inconsistent with the types of malformations induced by artemisinins in animal studies.

These results have important implications for malaria treatment and control policies, and future studies of artemisinin safety. Recurrent first-trimester vivax malaria is associated with miscarriage, yet radical cure is not possible during pregnancy with currently available drugs. First-trimester falciparum malaria is strongly associated with miscarriage, especially after recurrence. We noted no evidence of harm associated with first-line artemisinin treatment of first-trimester falciparum malaria. Quinine is comparatively poorly tolerated and associated with a shorter time to recurrence than artemisinin in pregnant women.^30^ Furthermore, women who received artemisinins following quinine failure were more likely to miscarry than those who received first-line artemisinin treatment. Early and effective antimalarial treatment is imperative, especially because current preventive measures do not adequately cover early pregnancy.^3^ Artemisinins are the most effective antimalarials available and have been recommended as first-line treatment in the general population by the WHO since 2006. Yet, artemisinin safety in first trimester is still a concern. This study contributes a further 183 well-documented first-trimester artemisinin treatments, and adds to a growing body of observational evidence supporting the use of artemisinins in the first trimester of pregnancy.^3^, ^11^, ^12^, ^14^, ^15^, ^16^, ^17^, ^18^, ^22^, ^31^ In view of the wide availability of artemisinin-based combination therapies, their excellent tolerability and efficacy, the likely reduced future availability of quinine, and the rarity of congenital malformations, now might be the time to endorse the use of artemisinin derivatives for the treatment of first-trimester falciparum malaria, accompanied by robust pharmacovigilance.

**This online publication has been corrected. The corrected version first appeared at thelancet.com/infection on April 18, 2016**

## Figures and Tables

**Table 1:** Cohort demographics

			**No first-trimester malaria (n=22 927)**	**First-trimester malaria (n=2558)**
Miscarried*			1963 (9%)	294 (14%)
Lost to follow-up before 28 weeks' gestation†			1949 (9%)	418 (16%)
Gestation at first consultation (weeks)			9·0 (7·2–11·3) [0·0–14·0]	8·4 (6·6–10·6) [0·1–14·0]
Maternal age (years)			26 (21–31) [13–51]	23 (19–30) [13–46]
	13–20		5443 (24%)	909 (36%)
	21–25		5959 (26%)	628 (25%)
	26–30		5709 (25%)	482 (19%)
	≥31		5816 (25%)	539 (21%)
Primigravid			5628 (25%)	821 (32%)
Current smoker			5362 (26%)	764 (35%)
History of miscarriage			6200 (27%)	758 (30%)
Haematocrit (first consultation; %)			36 (33–38) [9–52]	34 (31–37) [13–48]
	Severe anaemia (haematocrit <20%)		9 (0%)	13 (1%)
Non-malaria febrile morbidity in first trimester			310 (1%)	38 (1%)
Number of antenatal malaria screens			23 (14–28) [1–40]	22 (15–28) [1–38]
Estimated gestational age from ultrasonography scans			16 714 (73%)	1648 (64%)
Details of initial first-trimester malaria				
Symptoms				
	Asymptomatic		NA	919 (36%)
	Symptomatic		NA	1639 (64%)
First-line treatment of first-trimester falciparum malaria				
	Quinine		NA	971 (81%)
	Mefloquine monotherapy		NA	25 (2%)
	Artemisinin derivative		NA	183 (15%)
		Mefloquine–artesunate	NA	71 (6%)
		Artemether–lumefantrine	NA	10 (1%)
		Artesunate plus clindamycin	NA	50 (4%)
		Artesunate monotherapy	NA	49 (4%)
		Dihydroartemisinin–piperaquine	NA	3 (0%)
	Other or unknown		NA	8 (1%)
	Died before administration		NA	20 (2%)

Data are median (IQR) [range] or n (%). Missing: gravidity, ten; smoking status, 2853; history of miscarriage, nine; haematocrit, 969; and miscarriage, 2367 (ie, lost to follow up before 28 weeks' gestation). Continuous variables were compared between groups using the Student's *t* test for normal distribution or the Mann-Whitney *U* test for skewed distribution. Categorical variables were compared with the χ^2^ test.

* In women followed until 28 weeks; women who miscarried presented for antenatal care 1·4 weeks earlier (p<0·0001), because early attendance increases the chances that a miscarriage will be detected.

† Women lost to follow-up were slightly younger (p<0·0001), and were more likely to be primigravid (p<0·0001) and have first-trimester malaria (p<0·0001; appendix p 6). NA=not applicable.

**Table 2:** Major congenital malformations by first-line treatment of first-trimester falciparum malaria by organ system

	**No malaria (n=18 803)**	Uncomplicated falciparum malaria (n=773)*		Hyperparasitaemic or severe falciparum malaria (n=31)*	
		Quinine (n=641)	Artemisinin (n=109)	Quinine (n=8)	Artemisinin (n=22)
Multiple	26 (17%)	1 (13%)	0	0	0
Syndromic	4 (3%)	0	0	0	0
CNS	33 (21%)	1 (13%)†	0	0	0
Ears, eyes, face, or neck	24 (15%)	0	1 (50%)‡	0	0
Circulatory	13 (8%)	1 (13%)§	0	0	0
Respiratory	2 (1%)	0	0	0	0
Digestive	59 (37%)	2¶ (25%)	1‖ (50%)	0	1** (50%)
Genital	19 (12%)	0	0	0	0
Renal	6 (4%)	0	0	0	0
Musculoskeletal	42 (27%)	4†† (50%)	0	0	1‡‡ (50%)
Skin	4 (3%)	0	0	0	0
Other	1 (1%)	0	0	0	0

Data are n (%).

* 24 women received first-line mefloquine or had unknown first-line treatment (zero malformations).

† Anencephaly (treated at 10 weeks' gestation).

‡ Microphthalmia (8 weeks').

§ Heart defect (7 weeks').

¶ Cleft lip and palate (10 weeks'); cleft lip and palate (9 weeks').

‖ Imperforated anus (6 weeks').

** Cleft lip and palate (14 weeks').

†† Polydactyly (12 weeks'); polydactyly (12 weeks'); syndactyly and talipes (11 weeks'); syndactyly (9 weeks').

‡‡ Syndactyly (13 weeks').
