# Corrections

**DOI:** 10.1016/S1473-3099(16)30014-7

**Published:** 2016-05

*Moore K A, Simpson J A, Pawet M K, et al. Safety of artemisinins in first trimester of prospectively followed pregnancies: an observational study.* Lancet Infect Dis *2016; **16:** 576–83*—In figure 4, the footnote symbols in the legend should have read ‘*Hazard ratios are shown for treatments occurring before (<6 weeks' gestation) and during (≥6 and <13 weeks' gestation) the embryosensitive window. †Artemisinin rescue after quinine failure refers to artemisinin-based treatment in first trimester following failure of first-line treatment with quinine or quinine plus clindamycin.’ These corrections have been made to the online version as of April 18, 2016; the print version is correct.

