# Supplementary material for: Safety of artemisinins in first trimester of prospectively followed pregnancies: an observational study
Source: Lancet Infect Dis. 2016 May;16(5):576–83. doi: 10.1016/S1473-3099(15)00547-2 (PMC4835584; doi:10.1016/S1473-3099(15)00547-2)
Supplement: Supplementary appendix [file mmc1.pdf]

THE LANCET Infectious Diseases

Supplementary webappendix

This webappendix formed part of the original submission and has been peer reviewed. We post it as supplied by the authors.

Supplement to: Moore KA, Simpson JA, Paw MK, et al. Safety of artemisinins in first trimester of prospectively followed pregnancies: an observational study. *Lancet Infect Dis* 2016; published online Feb 8. http://dx.doi.org/10.1016/S1473-3099(15)00547-2.

Web Extra Material for: "The safety of artemisinins in first trimester in prospectively followed pregnancies: an observational study."

Systematic search

We searched SCOPUS and PubMed for articles published up to October 5 2015 (in any language) addressing the association between artemisinin treatment during first trimester and miscarriage using the search terms: "malaria or plasmodium", "pregnan*", "*artemisinin* OR ACT* OR artesunate OR artemether OR Coartem", "first-trimester OR 'first trimester'", and "miscarriage* OR abortion". Identified studies from this search are shown in Table 1.

Table 1. Identified published articles reporting on the association between use of artemisinin derivatives in the first trimester of pregnancy and miscarriage and/or congenital malformations

Reference	Country	First-trimester treatments	Artemisinin derivative	Association with miscarriage	Association with congenital abnormality
Dellicour 2015[1]	Kenya	299	Unspecified ACT	No	-
Poespoprodjo 2014[2]	Indonesia	8	DHP	Yes	No
Mosha 2014[3]	Tanzania	172	AL	No	No
Dellicour 2013[4]	Senegal	7	ACT	No	No
Rulisa 2012[5]	Rwanda	96	AL	Yes	No
Manyando 2010[6,7]	Zambia	159	AL	Unclear	No
Adam 2009[8]	Sudan	62	AS, AS-SP, AL	No	No
Adam 2004[9]	Sudan	1	Artemether	No	No
Deen 2001[10]	Gambia	77	AS with SP	No	No
Willcox 2011[11]	Kenya, Uganda	54	*Artemisia annua*	No	-
**TOTAL***		**935**			
**Systematic Reviews**					
Dellicour 2007[12]	Systematic review	123	[Review]	No	Inconclusive
**SMRU studies**					
McGready 2012[13]		44	AS, AC, MAS, AL.	No	No
McGready 2001[14]		44	AS, Artemether	No	No
McGready 2003[15]		3	AAP	-	No
Rijken 2008[16]		3	DHP	-	-

Abbreviations: ART = artemisinin; AL = artemether-lumefantrine (Coartem); AS = artesunate; SP = sulfadoxine-pyrimethamine; ACT = artemisinin-combination therapy; AC = artesunate plus clindamycin; MAS = mefloquine artesunate; AAP = Artesunate-atovaquone-proguanil; DHP = dihydroartemisinin-piperaquine. *Total does not include SMRU studies since the current analysis re-analyses these treatments[12].

References for Table 1:

1 Dellicour S, Desai M, Aol G, *et al.* Risks of miscarriage and inadvertent exposure to artemisinin derivatives in the first trimester of pregnancy: a prospective cohort study in western Kenya. *Malar J* 2015; **14**. DOI:10.1186/s12936-015-0950-6.

2 Poespoprodjo JR, Fobia W, Kenangalem E, *et al.* Dihydroartemisinin-piperaquine treatment of multidrug resistant falciparum and vivax malaria in pregnancy. *PLoS One* 2014; **9**: e84976.

3 Mosha D, Mazuguni F, Mrema S, Sevene E, Abdulla S, Genton B. Safety of artemether-lumefantrine exposure in first trimester of pregnancy: an observational cohort. *Malar J* 2014; **13**: 197.

4 Dellicour S, Brasseur P, Thorn P, *et al.* Probabilistic record linkage for monitoring the safety of artemisinin-based combination therapy in the first trimester of pregnancy in Senegal. *Drug Saf* 2013; **36**: 505–13.

5 Rulisa S, Kaligirwa N, Agaba S, Karema C, Mens PF, Vries PJ De. Pharmacovigilance of artemether-lumefantrine in pregnant women followed until delivery in Rwanda. *Malar J* 2012; **11**.

6 Manyando C, Mkandawire R, Puma L, *et al.* Safety of artemether-lumefantrine in pregnant women with malaria: results of a prospective cohort study in Zambia. *Malar J* 2010; **9**.

7 Manyando C, Njunju EM, Virtanen M, Hamed K, Gomes M, Van geertruyden J-P. Exposure to

artemether-lumefantrine (Coartem®) in first trimester pregnancy in an observational study in Zambia. *Malar J* 2015; **14**: 77.

8 Adam I, Elhassan EM, Omer EM, Abdulla M a, Mahgoub HM, Adam GK. Safety of artemisinins during early pregnancy, assessed in 62 Sudanese women. *Ann Trop Med Parasitol* 2009; **103**: 205–10.

9 Adam I, Elwasila E, Alnour D, Ali M, Elansari E, Idris M. Artemether in the treatment of falciparum malaria during pregnancy in eastern Sudan. *Trans R Soc Trop Med Hyg* 2004; **98**: 509–13.

10 Deen J, Seidlein L von, Pinder M, Walraven G, Greenwood B. The safety of the combination during pregnancy artesunate and pyrimethamine-sulfadoxine given. *Trans R Soc Trop Med Hyg* 2001; **95**: 424–8.

11 Willcox ML, Burton S, Oyweka R, Namyalo R, Challand S, Lindsey K. Evaluation and pharmacovigilance of projects promoting cultivation and local use of Artemisia annua for malaria. *Malar J* 2011; **10**: 84.

12 Dellicour S, Hall S, Chandramohan D, Greenwood B. The safety of artemisinins during pregnancy: a pressing question. *Malar J* 2007; **6**. DOI:10.1186/1475-2875-6-15.

13 McGready R, Lee SJ, Wiladphaingern J, *et al.* Adverse effects of falciparum and vivax malaria and the safety of antimalarial treatment in early pregnancy: a population-based study. *Lancet Infect Dis* 2011; published online Dec. DOI:10.1016/S1473-3099(11)70339-5.

14 McGready R, Cho T, Keo NK, *et al.* Artemisinin antimalarials in pregnancy: a prospective treatment study of 539 episodes of multidrug-resistant Plasmodium falciparum. *CID* 2001; **33**: 2009–16.

15 McGready R, Keo NK, Villegas L, White NJ, Looareesuwan S, Nosten F. Aresunate-atovaquone-proguanil rescue treatment of multidrug-resistant Plasmodium falciparum malaria in pregnancy: a preliminary report. *Trans R Soc Trop Med Hyg* 2003; **97**: 592–4.

16 Rijken MJ, McGready R, Boel ME, *et al.* Dihydroartemisinin-piperaquine rescue treatment of multidrug-resistant Plasmodium falciparum malaria in pregnancy: a preliminary report. *Am J Trop Med Hyg* 2008; **78**: 543–5.

Table 2. Procedures - extended

Procedure	Extension to Procedures in main text
Estimating gestational age	Prior to ultrasound, last menstrual period, symphysis-pubis fundal height measurement, or the Dubowitz gestational age assessment were used to estimate gestational age, and clinical signs were relied upon to determine fetal viability:<br><br>Methods for estimating gestational age at SMRU clinics:<br>• Last menstrual period (all years)<br>• Symphysis-pubis fundal height (SFH) measurement (1986 – 1994)[17]<br>• The Dubowitz gestational age assessment (1992 – 2002)<br>• Ultrasound biometry (2001 – present)[18]<br><br>Although conception does not occur until two weeks gestation, we included women from zero weeks gestation since their pregnancy was later confirmed and methods for estimating gestational age tend to overestimate in this population.[19]
Determination of fetal viability	Signs of fetal non-viability:<br>• Clinical signs (prior to ultrasound): significant vaginal bleeding and pain, an open cervix, expulsion of products of conception, or a negative pregnancy test.<br>• Ultrasound examination: absence of a fetal heartbeat, an anembryonic gestation, retained products of conception, an ectopic pregnancy, or a hydatidiform mole. Ultrasound determination of viability improved after 2007 with better training, data recording, and ultrasound equipment at all sites.
Screening frequency	Screening was initially weekly but less frequent in later years to avoid false positives associated with decreasing transmission. In the migrant communities, screening switched from weekly to fortnightly in 2010. In the refugee camps, starting in 2007, women were screened weekly for the first three weeks of antenatal care, and weekly screening continued only if one or more of these three screens were positive. If all three screens were negative women were only screened again if they slept outside the camp or presented with fever.
Malaria screening and definition	Finger-prick blood samples are examined by trained microscopists using Giemsa stained thick and thin blood films. Plasmodia parasites are counted per 500 white blood cells of 1000 red blood cells.
Definition of major congenital malformations	Major congenital malformations were defined as a structural abnormality with surgical, medical, or cosmetic importance ascertained at birth. Birthmarks, minor physical features (e.g. preauricular pits/sinus), normal variations, and positional deformities not due to a primary problem in morphogenesis were not considered.

Table 3. Treatment regimens according to type of *P. falciparum* infection

Type of infection [Total artemisinin]	Treatment regimen [manufacturer[1]]
First-trimester, uncomplicated [0 mg/kg]	**Quinine**[2], 10 mg salt/kg three times/day for 7 days [Government Pharmaceutical Organization, Thailand]
Recrudescent [12-14mg/kg]	**Artesunate**[2,3] 2 mg/kg on days 0-4 & 1 mg/kg on days 5-6<br>OR<br>**Artesunate**[2,3] 4 mg/kg on day 0 & 2 mg/kg on days 1-2 & 1 mg/kg on days 3- 6<br>OR<br>**Artesunate**[2,3] 2 mg/kg/day over 7 days<br>OR<br>**Artesunate** 4 mg/kg per day for 3 days plus **Malarone**™ [Glaxo-Wellcome] at 20 mg/kg/day atovaquone plus 8 mg/kg/day proguanil for 3 days<br>OR<br>**Dihydroartemisinin-piperaquine** (dihydroartemisinin 3 mg/kg, piperaquine 15-17 mg/kg [Holley Pharmaceuticals]) once daily for 3 days<br>OR<br>**Quinine**[2], 10 mg salt/kg three times/day for 7 days [Government Pharmaceutical Organization, Thailand], especially when still in the first trimester
Hyperparasitaemia[5] [12-16mg/kg]	**Artesunate**[2,3] 4 mg/kg on day 0 & 2 mg/kg on days 1-2 & 1 mg/kg on days 3- 6<br>OR<br>**Artesunate**[2,3] 4 mg/kg on day 0 & 2 mg/kg on days 1-6
Severe[5] [12-16mg/kg]<br><br>[0 mg/kg]	**Artesunate**[2] 4 mg/kg loading dose (or intramuscular artemether 3.2 mg/kg[5]) followed by artesunate routine dose of 2 mg/kg/day for 6 days.<br>OR<br>Intravenous **artesunate**[2] 2.4 mg/kg loading dose followed by 1.2 mg/kg at 12 h and 24 h, and repeated every 24 h until oral intake possible, then oral artesunate 2 mg/kg/day for a total of 7 day treatment.<br>OR<br>Intravenous **artesunate**[2] 2.4 mg/kg at baseline, then 12 and 24 hrs and every 24 hrs until oral intake possible, then oral artesunate 2 mg/kg/day, for a total of 7 day treatment.<br>OR<br>Intravenous **quinine**[2] 20mg/kg loading dose over 4 hours, followed by 10mg/kg over 2 hours, every 8 hours until oral intake tolerated, then oral quinine 10 mgkg oral every hours (or available ACT), for a total of 7 day treatment.
Inadvertent[7] [12-14 mg/kg]	**Artesunate**[4], as a single daily dose of 4 mg/kg over 3 days with **mefloquine**, 25 mg/kg given as a split regimen, 24 h (15 mg/kg) and 48 h (10 mg/kg) or 8/mg/day for 3 days<br>OR<br>**Artesunate**[2], 2 mg/kg/ day for 7 days<br>OR<br>**Artemether-lumefantrine**, 4 tablets (20 mg artemether plus 120 mg lumefantrine) twice daily for 3 days [Novartis]<br>OR<br>**Dihydroartemisinin-piperaquine**, DHA 3mg/kg plus PPQ 15-17 mg/kg per day for 3 days [Holley Pharmaceuticals]<br>OR<br>**Mefloquine monotherapy** 25 mg/kg stat of given as a split regimen, 24 h (15 mg/kg) and 48 h (10 mg/kg)<br>OR<br>**Artesunate**, 2 mg/kg/day for 7 days and **doxycycline**[6] 200 mg OD for 7 days

[1]Artesunate manufacturer: Guilin Pharmaceutical; mefloquine manufacturer: Roche, Switzerland/CIPLA, India. [2]Alone or in combination with clindamycin [Siam Bheasach/Pharmacia & Upjohn] at 5 mg/kg/dose every 8 hours for 7 days. [3]Alone or in combination with: mefloquine 25 mg/kg given as a split regimen, 24 h (15 mg/kg) and 48 h (10 mg/kg). [4]The standard 1st line treatment for non-pregnant patients with uncomplicated malaria. [5]In later years an IM dose of artemether (1.6mg/kg) was used if hyperparasitaemic cases showed slow parasite clearance (>95th centile) in the first 24 hours after oral artesunate and this was changed to 1.2mg/kg artesunate when absorption of artemether was recognized as problematic in some unwell patients. [6]Non-pregnant patients treated with doxycycline to avoid mefloquine because they already had it in previous 63 days (they were recrudescent cases) as doxycycline 200 mg daily for the same number of days as artesunate. SMRU malaria treatment guidelines are available online: http://www.shoklo-unit.com/index.php/malaria-guideline.

Table 4. Statistical methods – extended

Statistical concept/method	Explanation
Left truncation[20,21]	Our data is left truncated because only pregnancies that survived until the first antenatal consultation can be detected and included in the analysis, while an unknown number of women miscarry before they ever present. That is, observation does not begin until sometime after women become at risk of miscarriage. Additionally, the risk of miscarriage decreases dramatically as a pregnancy progresses, so that women who present later are less likely to miscarry than those who present earlier. Therefore, entry is differential by outcome (miscarriage), and also by exposure (those with malaria are prompted to present earlier, and the median gestational age is different across first-line treatments). This will induce a bias unless left truncation is accounted for using survival analysis with gestation time as the time scale and only allowing women to enter the analysis when they came under observation (i.e. at the first antenatal consultation), rather than when they became at risk of miscarriage (i.e. at conception).
Confounding by indication and disease severity[22]	Confounding by indication – *P. falciparum* infection (the indication) is associated with both antimalarial treatment and miscarriage, thereby confounding the association between treatment and miscarriage. Confounding by indication can be avoided by only including women who had *P. falciparum* (i.e. all have the same indication) and comparing the treatment of interest (i.e. artemisinin) to a treatment that is considered not to be associated with the outcome (i.e. quinine).<br><br>Confounding by disease severity – the severity of *P. falciparum* infection is associated with both the antimalarial administered and miscarriage. For example, artemisinins are recommended for first-line treatment of severe or hyperparasitaemic *P. falciparum* infections in first trimester, so artemisinins are more likely to be given in these cases than quinine, but severe or hyperparasitaemic disease is also more likely to cause miscarriage than uncomplicated infections. This can be avoided by adjusting for severity.
Right censoring	Cox regression assumes non-informative censoring, but it is possible that there was an association between loss to follow-up before 28 weeks and miscarriage (i.e. informative right censoring), since women who miscarry may not return to antenatal care, especially if they miscarry early in pregnancy.<br><br>A higher proportion of women lost to follow-up had malaria in first trimester (Table 1). However, less than 10% of pregnancies were lost to follow-up (Table 1), and only 5% were lost before 14 weeks gestation, after which the risk of miscarriage is extremely low and women are more likely to seek medical care. Informative right censoring would underestimate the association between malaria and miscarriage. We consider an underestimation of the association between malaria and miscarriage to be more tolerable than an overestimation, as we can be confident that the source of bias has not produced a false association.<br><br>Most (70; 96%) pregnancies that received first-line MAS were retained, compared to 80% of women who received first-line quinine. Therefore, it is possible that the association between artemisinin treatment and miscarriage has been overestimated. We consider an overestimation of the association between artemisinin treatment and miscarriage to be more tolerable, as we can be confident that the source of bias has not hidden an association between artemisinin treatment and miscarriage.

Table 5. Demographics of included and excluded pregnancies

Characteristic	Excluded, N = 30151	Included, N = 25485	P value
Gestation at first consultation, weeks	21.2 {16.5, 28.0}, 2 – 42	9.0 {7.1, 11.3}, 0 – 14.0	<0.0001
Age, years	26 {21 – 31}, 13 – 53	25 {21 – 30}, 13 – 51	<0.0001
Primigravidae	8114 (27)	6449 (25)	<0.0001
Smoker	6936 (27)	6126 (27)	0.3434
History of miscarriage	7256 (24)	6958 (27)	<0.0001
Haematocrit at first ANC, %	33 {30 – 35}, 8 – 53	35 {33 – 38}, 9 – 52	<0.0001

Missing data: gestation 1490; age 95; gravidity 83; smoking status 7740; history of miscarriage 76; haematocrit 4242.

Table 6. Demographics of pregnancies lost to follow-up before 28 weeks gestation

Characteristic	Lost, N = 2367	Retained, N = 23118	P value
Gestation at first consultation, weeks	9.0 {7, 11.3}, 0 – 14.0	9.0 {7.2, 11.3}, 0 – 14.0	
Age, years	24 {20 – 30}, 14 – 46	25 {21 – 31}, 13 – 51	<0.0001
Primigravidae	798 (34)	5651 (24)	<0.0001
Smoker	575 (26)	5551 (27)	0.1136
Malaria in 1st trimester	417 (18)	2140 (9)	<0.0001
Non-malaria febrile morbidity in 1st trimester	42 (2)	306 (2)	0.0719
History of miscarriage	638 (30)	6320 (28)	0.6814
Haematocrit, %	35 {33 – 38}, 9 – 49	35 {33 – 38}, 12 – 52	<0.0001

Missing data: age 4; gravidity 10; smoking status 2853; history of miscarriage 9; haematocrit 969.

Table 7. Lost to follow-up before 28 weeks gestation in 1179 women with first-trimester falciparum malaria by first-line treatment

First-line treatment	Lost, N = 215	Retained, N = 972
Artemisinin	25 (14)	158 (86)
Mefloquine-artesunate*	4 (6)	67 (94)
Other artemisinins	21 (19)	91 (81)
Mefloquine monotherapy	4 (16)	21 (84)
Quinine	186 (19)	785 (81)

Excludes women treated after determination of non-viability. *P*-value with and without mefloquine-artesunate breakdown 0.0417 and 0.2016, respectively. *Women who were inadvertently treated with mefloquine artesunate in first trimester at outpatient clinics were closely followed by SMRU, resulting in higher retention.

Table 8. Antimalarial drug treatments and species by order of first-trimester falciparum malaria episode in 1207 pregnancies with first-trimester falciparum malaria

Infection	ART	Q	MFQ	Demised[1]	Other[2]
1st falciparum malaria episode, N = 1207	183 (15)*	971 (80)**	25 (2)	20 (2)	8 (1)
2nd falciparum malaria episode, N = 162	97 (60)**	45 (28)	4 (2)	10 (6)	6 (4)
3rd falciparum malaria episode, N = 12	5 (42)	7 (58)	0 (0)	0 (0)	0 (0)
4th falciparum malaria episode, N = 1	1 (100)	0 (0)	0 (0)	0 (0)	0 (0)

Abbreviations: ART – artemisinin-based treatment; Q – quinine-based treatment; MFQ – mefloquine monotherapy.[1]Antimalarial treatment administered after determination of non-viability. [2]1st falciparum malaria episode: one doxycycline treatment, one atovaquone-proguanil treatment, two chloroquine treatments, two unknown treatments, and two women not treated; 2nd *Pf*: five doxycycline treatments and one woman not treated. *37 first-line artemisinin treatments were given for hyperparasitaemia or severe disease; five women received a second artemisinin treatment for a subsequent first-trimester falciparum malaria episode (4 artesunate, 1 artesunate plus clindamycin, and 1 artesunate plus doxycycline). **129 women received ART after first-line quinine failure, including 37 women that were rescued with ART within a week of starting first-line Q treatment (counted as Q treatment of 1st *Pf* infection), and 92 women who received ART for a subsequent falciparum malaria episode after first-line quinine treatment (counted in Table 3 as ART treatment of 2nd falciparum malaria episode).

Table 9. Risk of miscarriage by first-line artemisinin-based treatment in first-trimester

Artemisinin-based treatment	Delivered, N (%)	Miscarried, N (%)
Mefloquine artesunate	56 (79)	15 (21)
Artemether-lumefantrine	10 (100)	0 (0)
Artemisinin plus clindamycin	48 (96)	2 (4)
Artesunate monotherapy	42 (86)	7 (14)
Dihydroartemisinin piperaquine	3 (100)	0 (0)

Table 10. Characteristics by miscarriage and first-line antimalarial treatment for first-trimester falciparum malaria

		All	p^1	Q or Q+C	Q Rescue	MFQ	MAS	Other ART	p^2
**Number (%)**	**All**	**1026**	**0.066**	**842**	**129**	**25**	**71**	**118**	
	**Mis.**	**153**		**104 (12)**	**22 (17)**	**3 (12)**	**15 (21)**	**9 (8)**	
	**Del.**	**1179**		**738 (88)**	**107 (83)**	**22 (88)**	**56 (79)**	**103 (92)**	
**Initial first-trimester *P. falciparum* infection**									
EGA at treatment	All	9.8 {6.7, 11.9}, 0.0 – 14.0	<0.001	10.1 {7.5, 11.8}, 0.0 – 14.0	7.4 {6.0, 10.3}, 2.5 – 13.7	8.4 {4.2, 13.0} 0.4 – 13.9	3.8 {1.9, 7.4}, 0.0 – 13.0	12.4 {9.0, 13.3}, 0.2 – 13.9	<0.001
	Mis.	7.0 {4.7, 9.3}, 0.0 – 13.9		7.7 {5.0, 10.2}, 2.0 – 13.9	5.6 {4.0, 7.8}, 2.8 – 11.9	4.7{2.6, 8.4}, 2.6 – 8.4	4.2 {0.5, 7.4}, 0.0 – 11.0	6.9 {5.9, 9.0}, 4.3 – 13.0	0.079
	Del.	10.1 {7.2, 12.0}, 0.0 – 14.0		10.3 {8,0, 11.9}, 0.0 – 14.0	7.9 {6.2, 10.6}, 2.5 – 13.7	10.2 {4.2, 13.1}, 0.4 – 13.9	3.7 {2.1, 7.4}, 0.1 – 13.0	12.5 {9.8, 13.3}, 0.2 – 13.9	<0.001
Symptomatic	All	858 (73)	<0.001	613 (73)	96 (74)	23 (92)	68 (96)	58 (52)	<0.001
	Mis.	123 (80)		87 (84)	17 (77)	3 (100)	15 (100)	1 (11)	<0.001
	Del.	735 (72)		526 (71)	79 (74)	20 (91)	53 (95)	57 (55)	<0.001
Hyperparasitaemic or severe	All	54 (5)	<0.001	8 (1)	9 (7)	0 (0)	1 (1)	36 (32)	<0.001
	Mis.	15 (10)		4 (4)	3 (14)	0 (0)	0 (0)	8 (89)	<0.001
	Del.	39 (4)		4 (1)	6 (6)	0 (0)	1 (2)	28 (27)	<0.001
Parasitaemia, µL[4]	All	1022 (876, 1194)	<0.001	750 (630, 892)	2771 (1767, 4345)	278 (124, 625)	984 (470, 2061)	3803 (2158, 6700)	<0.001
	Mis.	5026 (3307, 7640)		3120 (1966, 4953)	15210 (6196, 37340)	No observations	969 (150, 6241)	205705 (121379, 348615)	<0.001
	Del.	814 (692, 957)		614 (512, 737)	1912 (1173, 3115)	279 (124, 625)	988 (424, 2301)	2655 (1510, 4669)	<0.001
Primigravid	All	346 (29)	0.351	255 (30)	37 (29)	6 (24)	9 (13)	39 (35)	0.018
	Mis.	40 (26)		28 (27)	7 (32)	0 (0)	2 (13)	3 (33)	0.562
	Del.	306 (30)		227 (31)	30 (28)	6 (27)	7 (13)	36 (35)	0.043
Refugee	All	376 (32)	0.312	276 (33)	24 (19)	21 (84)	32 (45)	23 (21)	<0.001
	Mis.	57 (37)		41 (39)	3 (14)	3 (100)	7 (47)	3 (33)	0.050
	Del.	319 (31)		235 (32)	21 (20)	18 (82)	25 (45)	20 (19)	<0.001
**Recurrent *P. falciparum* infections**									
Before 14 weeks gestation	All	159 (13)	0.004	47 (6)	96 (74)	3 (12)	9 (13)	4 (4)	<0.001
	Mis.	32 (21)		12 (12)	15 (68)	2 (67)	2 (13)	1 (11)	<0.001
	Del.	127 (12)		35 (5)	81 (76)	1 (5)	7 (13)	3 (3)	<0.001
Before 28 weeks gestation	All	374 (32)	0.050	229 (27)	102 (79)	7 (28)	15 (21)	21 (19)	<0.001
	Mis.	38 (25)		17 (16)	15 (68)	2 (67)	3 (20)	1 (11)	<0.001
	Del.	336 (33)		212 (29)	87 (81)	5 (23)	12 (21)	20 (19)	<0.001

Numbers are median {25th – 75th percentile}, range, or N (%). [1]For the association between variable and miscarriage. [2]For the association between variable and first-line treatment within all women, women who miscarried, or women who delivered. [4]Geometric mean (95% CI); Parasitaemia missing in 9 (Other ART), 29 (MAS), 10 (MFQ), 6 (Q rescue), and 53 (Q or Q+C). [4]Parasitaemia was not included in multivariable models because of missing values, adjustment did not change estimates by more than 10%, and parasitaemia is strongly associated with symptoms (*p* <0.001). [5]Three women received a second first-trimester ART treatment (two artesunate monotherapy and one artesunate doxycycline) after first-line MAS, and all delivered. [6]Four women received a second first-trimester ART treatment (three artesunate monotherapy and one artesunate plus clindamycin) after first-line ART, and one miscarried.

Table 11. The association between initial and recurrent first-trimester malaria and miscarriage in women with no first-trimester *P. vivax* infections

Model/Model parameter	Delivered	Miscarried	Total weeks at risk	Unadjusted HR (95% CI); p-value	Adjusted[6] HR (95% CI); p-value
1: Falciparum malaria (all)					
No malaria	18995 (91)	1963 (9)	379716	Ref.	Ref.
Initial	591 (84)	115 (16)	12533	1.74 (1.44, 2.10); <0.001	1.74 (1.41, 2.16); <0.001
Recurrent	85 (77)	25 (23)	1632	3.36 (2.27, 4.99); <0.001	3.49 (2.31, 5.28); <0.001
2: Asymptomatic falciparum					
No malaria	18995 (91)	1963 (9)	379009	Ref.	Ref.
Initial	161 (91)	15 (9)	3026	1.14 (0.68, 1.89); 0.621	1.45 (0.87, 2.42); 0.150
3: Symptomatic falciparum					
No malaria	18995 (91)	1963 (9)	379604	Ref.	Ref.
Initial	430 (81)	100 (19)	9357	1.95 (1.60, 2.39); <0.001	1.89 (1.50, 2.38); <0.001
Recurrent	45 (67)	22 (33)	915	5.02 (3.30, 7.65); <0.001	5.20 (3.34, 8.10); <0.001

Numbers are hazard ratios (HR) (95% confidence interval); *p*-value. Cox models include women lost to follow-up before 28 weeks (until gestation time last seen), but percentage calculations for delivered/miscarried do not. Model 2 excludes pregnancies that had symptomatic infections. Model 3 excludes pregnancies that had asymptomatic infections. Models were adjusted for year, gravidity, current smoking status, and non-malaria febrile morbidity in first trimester.

Table 12. Table version of Figure 3 in main text: The association between initial and recurrent first-trimester malaria and miscarriage

Model/Model parameter	Delivered	Miscarried	Total weeks at risk	Unadjusted HR (95% CI); p-value	Adjusted HR (95% CI); p-value
1: Falciparum malaria (all)					
No malaria	18995 (91)	1963 (9)	380091	Ref.	Ref.
Initial	702 (84)	132 (16)	15049	1.64 (1.38, 1.96); <0.0001	1.61 (1.32, 1.97); <0.0001
Recurrent	113 (77)	33 (23)	2157	3.33 (2.36, 4.70); <0.0001	3.24 (2.24, 4.68); <0.0001
2: Asymptomatic falciparum					
No malaria	18995 (91)	1963 (9)	379099	Ref.	Ref.
Initial	165 (92)	15 (8)	3260	1.07 (0.64, 1.78); 0.7888	1.27 (0.76, 2.11); 0.3616
Recurrent	12 (92)	1 (8)	223	1.17 (0.17, 8.34); 0.8727	1.21 (0.17, 8.61); 0.8478
3: Symptomatic falciparum					
No malaria	18995 (91)	1963 (9)	379604	Ref.	Ref.
Initial	482 (82)	109 (18)	10429	1.89 (1.55, 2.29); <0.0001	1.83 (1.47, 2.28); <0.0001
Recurrent	54 (67)	27 (33)	1096	5.00 (3.42, 7.31); <0.0001	4.92 (3.28, 7.38); <0.0001
4: Hyper/severe falciparum					
No malaria	18995 (91)	1963 (9)	378818	Ref.	Ref.
Initial	31 (67)	15 (33)	712	3.89 (2.34, 6.47); <0.0001	4.21 (2.43, 7.29); <0.0001
5: Asymptomatic vivax					
No malaria	18995 (91)	1963 (9)	379682	Ref.	Ref.
Initial	479 (89)	58 (11)	9804	1.23 (0.95, 1.60); 0.1166	1.21 (0.89, 1.65); 0.2286
Recurrent	17 (100)	0 (0)	-	Omitted – no miscarriages	Omitted – no miscarriages
6: Symptomatic vivax					
No malaria	18995 (91)	1963 (9)	379458	Ref.	Ref.
Initial	454 (88)	63 (12)	9417	1.27 (0.99, 1.63); 0.0609	1.27 (0.96, 1.68); 0.0943
Recurrent	33 (87)	5 (13)	580	2.17 (0.90, 5.23); 0.0828	2.44 (1.01, 5.88); 0.0473
Year of 1st consultation	20837 (90)	2257 (10)	419903	0.94 (0.93, 0.95); <0.0001	(Stratified)
Gravidity					
Primigravid	5245 (93)	398 (7)	107057	Ref.	Ref.
Multigravid	15592 (89)	1859 (11)	312846	1.61 (1.44, 1.79); <0.0001	1.48 (1.30, 1.68); <0.0001
Maternal age					
13 – 20 years	5226 (92)	427 (8)	106552	Ref.	§
21 – 25 years	5453 (92)	456 (8)	110069	1.04 (0.91, 1.19); 0.5562	
26 – 30 years	5128 (91)	532 (10)	102828	1.31 (1.15, 1.48); <0.0001	
31+ years	5030 (86)	842 (14)	100456	2.14 (1.90, 2.40); <0.0001	
Current smoker					
No	13651 (92)	1172 (8)	275204	Ref.	Ref.
Yes	4860 (88)	685 (12)	98747	1.62 (1.48, 1.78); <0.0001	1.38 (1.25, 1.53); <0.0001
Previous miscarriage					
None	15335 (91)	1433 (9)	307398	Ref.	§
One or more	5493 (87)	824 (13)	112328	1.54 (1.41, 1.68); <0.0001	
Non-malaria febrile morbidity					
No	20596 (90)	2192 (10)	414529	Ref.	Ref.
Yes	241 (79)	65 (21)	5374	2.02 (1.58, 2.58); <0.0001	2.59 (1.99, 3.37); <0.0001

Numbers are adjusted hazard ratios (HR) (95% confidence interval) or N (%). Del. = delivered. Misc. = miscarried. Hyper = hyperparasitaemic. Cox models include women lost to follow-up before 28 weeks (until gestation time last seen), but percentage calculations for delivered/miscarried do not. Models for falciparum malaria (1 – 4) include women that may have also had first-trimester vivax, malariae, or ovale malaria; see webappendix Table 8 for associations in women with only first-trimester falciparum malaria. Models for vivax malaria (5 – 6) exclude women that also had first-trimester falciparum malaria. Models 2 and 5 exclude women with symptomatic malaria. Models 3 and 6 exclude women with asymptomatic infections. Model 4 excludes women with uncomplicated infections. Models were adjusted for year (by stratification due to non-proportional hazards (p <0.001)), gravidity, current smoking status, and non-malaria fever in first trimester. Age and previous miscarriage were omitted from multivariable models due to collinearity with gravidity. Adjusted results for gravidity, current smoking status, and fever in first trimester are presented from Model 1.

Table 13. Table version of Figure 4 in main text: The association between first-line treatment of first-trimester falciparum malaria and miscarriage, N = 1179

Treatment*	Embryo-sensitive window**	Delivered	Miscarried	Unadjusted HR	Adjusted HR
Q or Q + C		750 (89)	92 (11)	Ref.	Ref.
	Before	93 (76)	30 (24)	Ref. [Before]	Ref. [Before]
	During	585 (91)	57 (9)	Ref. [During]	Ref. [During]
	After	72 (94)	5 (6)	Ref. [After]	Ref. [After]
ART rescue		111 (86)	18 (14)	1.52 (0.92, 2.52); 0.1037	1.18 (0.67, 2.10); 0.5613
MFQ		23 (92)	2 (8)	0.61 (0.15, 2.47); 0.4881	0.54 (0.13, 2.31); 0.4082
ART		160 (87)	23 (13)	0.97 (0.61, 1.53); 0.8806	0.78 (0.45, 1.34); 0.3645
	Before	50 (82)	11 (18)	0.55 (0.28, 1.10); 0.0923	0.54 (0.25, 1.15); 0.1108
	During	64 (84)	12 (16)	1.80 (0.96, 3.35); 0.0648	1.15 (0.46, 2.87); 0.7602
	After	46 (100)	0 (0)	Omitted	Omitted

Numbers are hazard ratios (HR) (95% confidence interval); *p*-value. Adjusted for symptoms, non-malaria febrile morbidity in first trimester, and year of first consultation. *Categorisations are based on treatment of the first *Pf* infection (i.e. first-line treatment), except for "ART rescue" which refers to artemisinin-based treatment in first trimester following failure of first-line treatment with quinine or quinine plus clindamycin; Q = quinine; C = clindamycin; MFQ = mefloquine monotherapy; ART = artemisinin-based treatment (monotherapy or combination therapy). **Before: <6 weeks gestation; during: ≥6 & <13 weeks gestation; after: ≥13 & <14 weeks gestation.

Table 14. The association between first-line treatment of first-trimester falciparum malaria and miscarriage, excluding women with asymptomatic falciparum malaria, N = 919

Treatment*	Embryo-sensitive window**	Delivered	Miscarried	Unadjusted HR	Adjusted HR
Q or Q + C		550 (87)	80 (13)	Ref.	Ref.
	Before	86 (75)	29 (25)	Ref. [Before]	Ref. [Before]
	During	418 (89)	47 (10)	Ref. [During]	Ref. [During]
	After	46 (92)	4 (8)	Ref. [After]	Ref. [After]
ART rescue		87 (84)	16 (16)	0.56 (0.14, 2.27); 0.4148	0.55 (0.13, 2.34);0.4168
MFQ		21 (91)	2 (9)	1.44 (0.84, 2.46); 0.1834	1.27 (0.69, 2.32); 0.4418
ART		140 (86)	23 (14)	0.90 (0.56, 1.43); 0.6513	0.82 (0.48, 1.42); 0.4816
	Before	50 (82)	11 (18)	0.53 (0.26, 1.06); 0.0746	0.54 (0.25, 1.16); 0.1131
	During	56 (82)	12 (18)	1.72 (0.91, 3.24); 0.0953	1.20 (0.48, 3.00); 0.7014
	After	34 (100)	0 (0)	Omitted	Omitted

Numbers are hazard ratios (HR) (95% confidence interval); *p*-value. Adjusted for symptoms, non-malaria febrile morbidity in first trimester, and year of first consultation. *Categorisations are based on treatment of the first *Pf* infection (i.e. first-line treatment), except for "ART rescue" which refers to artemisinin-based treatment in first trimester following failure of first-line treatment with quinine or quinine plus clindamycin; Q = quinine; C = clindamycin; ART = artemisinin-based treatment (monotherapy or combination therapy). **Before: <6 weeks gestation; during: ≥6 & <13 weeks gestation; after: ≥13 & <14 weeks gestation.

Table 15. The association between first-line treatment of first-trimester falciparum malaria and miscarriage in pregnancies with ultrasound biometry between 2001 and 2007, N = 469

Treatment*	Embryo-sensitive window**	Delivered	Miscarried	Unadjusted HR	Adjusted HR
Q or Q + C		303 (93)	23 (7)	Ref.	Ref.
	Before	29 (81)	7 (19)	Ref. [Before]	Ref. [Before]
	During	238 (94)	15 (6)	Ref. [During]	Ref. [During]
	After	36 (97)	1 (3)	Ref. [After]	Ref. [After]
ART rescue		72 (88)	10 (12)	2.02 (0.96, 4.23); 0.064	1.78 (0.77, 4.14); 0.180
ART		55 (90)	6 (10)	1.17 (0.48, 2.88); 0.731	0.94 (0.33, 2.73); 0.913
	Before	19 (95)	1 (5)	0.21 (0.03, 1.73); 0.147	0.26 (0.03, 2.19); 0.214
	During	21 (81)	5 (19)	3.21 (1.16, 8.87); 0.024	4.31 (1.12, 16.56); 0.034
	After	15 (100)	0 (0)	Omitted	Omitted

Numbers are hazard ratios (HR) (95% confidence interval); *p*-value. Adjusted for symptoms, non-malaria febrile morbidity in first trimester, and year of first consultation. *Categorisations are based on treatment of the first *Pf* infection (i.e. first-line treatment), except for "ART rescue" which refers to artemisinin-based treatment in first trimester following failure of first-line treatment with quinine or quinine plus clindamycin; Q = quinine; C = clindamycin; ART = artemisinin-based treatment (monotherapy or combination therapy). **Before: <6 weeks gestation; during: ≥6 & <13 weeks gestation; after: ≥13 & <14 weeks gestation.

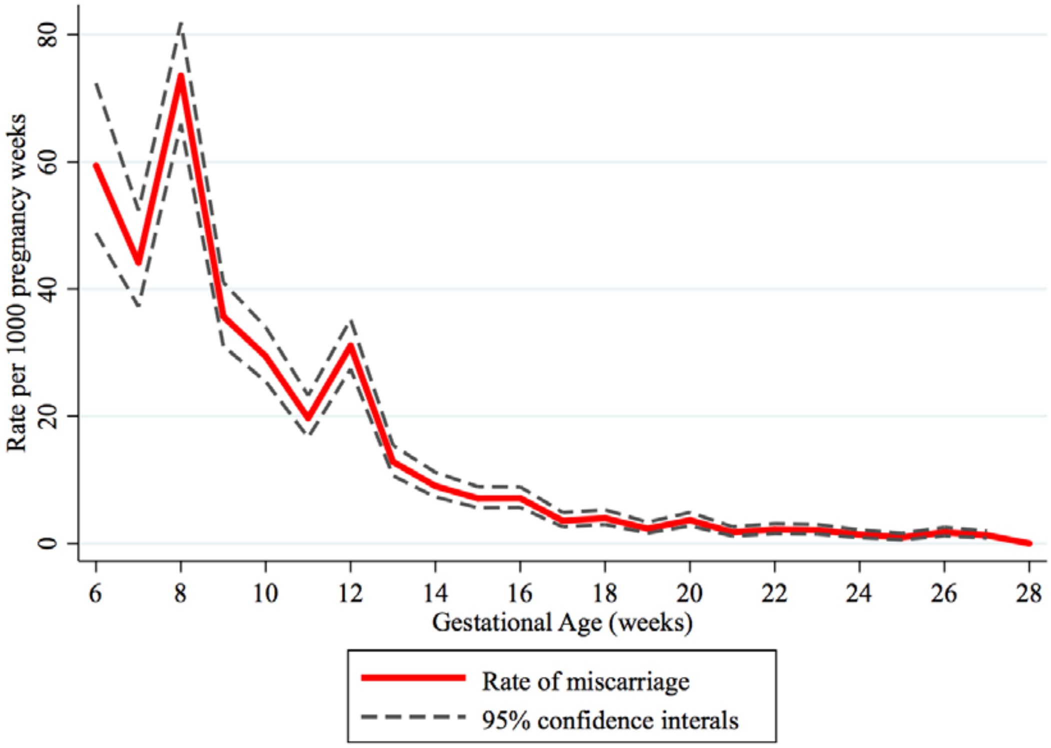

Figure 1. Rate of miscarriage over gestation time. Dotted grey lines are 95% confidence intervals. Rates are expressed per 1000 pregnancy weeks.

References

17 White LJ, Lee SJ, Stepniewska K, *et al*. Estimation of gestational age from fundal height: a solution for resource-poor settings. *J R Soc Interface* 2012; **9**: 503–10.

18 Rijken MJ, Lee SJ, Boel ME, *et al*. Obstetric ultrasound scanning by local health workers in a refugee camp on the Thai-Burmese border. *Ultrasound Obstet Gynecol* 2009; **34**: 395–403.

19 Moore KA, Simpson JA, Thomas KH, *et al*. Estimating gestational age in late presenters to antenatal care in a resource-limited setting on the Thai-Myanmar border. *PLoS One* 2015. DOI:10.1371/journal.pone.0131025.

20 Howards PP, Hertz-Picciotto I, Poole C. Conditions for bias from differential left truncation. *Am J Epidemiol* 2006; **165**: 444–52.

21 Meister R, Schaefer C. Statistical methods for estimating the probability of spontaneous abortion in observational studies-analyzing pregnancies exposed to coumarin derivatives. *Reprod Toxicol* 2008; **26**: 31–5.

22 Salas M, Hofman A, Stricker B. Confounding by indication: an example of variation in the use of epidemiologic terminology. *Am J Epidemiol* 1999; **149**: 981–3.
